# Period poverty, reuse needs, and depressive symptoms among refugee menstruators in Jordan’s camps: a cross-sectional study

**DOI:** 10.1186/s12905-024-03193-x

**Published:** 2024-07-03

**Authors:** Nadia Muhaidat, Joud Al Karmi, Abdulrahman M. Karam, Farah Abushaikha, Mohammad Ali Alshrouf

**Affiliations:** 1 Department of Obstetrics & Gynaecology, School of Medicine, The University of Jordan, Amman, 11942 Jordan; 2 The School of Medicine, The University of Jordan, Amman, 11942 Jordan; 3 Department of Urology, Abdali Hospital, Amman, 11191 Jordan

**Keywords:** Period poverty, Depressive symptoms, Menstrual poverty, Refugee, Menstruators, Menstrual health

## Abstract

**Background:**

Period poverty is a significant issue that impacts the physical and psychological well-being of menstruators worldwide which can further contribute to poor mental health outcomes. For menstruators living in refugee camps, access to menstrual hygiene products is often limited or non-existent, leading to increased anxiety, shame, and embarrassment. Therefore, this study aimed to assess the prevalence of the period poverty and to comprehensively analyze the association between period poverty, reusing menstrual products, and depressive symptoms among menstruators living in refugee camps in Jordan.

**Methods:**

A cross-sectional study surveyed refugee menstruators living in camps in Jordan, aged post-menarche to pre-menopause. Data collection included socio-demographics, menstrual practices, and depressive symptoms using the Patient Health Questionnaire (PHQ-9). Period poverty was assessed through affordability and frequency of struggles with menstrual products. Chi-squared test, independent sample t-test, One Way Analysis of variance (ANOVA) followed by Post hoc, and logistic regression models were used in the analysis.

**Results:**

The study included a diverse sample of 386 refugee menstruators living in camps in Jordan (mean age 32.43 ± 9.95, age range 13–55). Period poverty was highly prevalent, with 42.0% reporting monthly struggles to afford menstrual products, and 71.5% reusing menstrual products. Univariate analysis revealed that experiencing period poverty was significantly associated with a younger age of marriage, increased number of children, lower education level, lower mother and father education levels, unemployment, decreased monthly income, absence of health insurance, lower reuse need score, and increased PHQ-9 score (*p* < 0.05). Menstruators experiencing monthly period poverty were 2.224 times more likely to report moderate to severe depression compared to those without period poverty (95% CI 1.069–4.631, *P* = 0.033).

**Conclusion:**

This study highlights a significant association between period poverty and depressive symptoms among refugee menstruators in living in camps in Jordan, as high rates of period poverty were associated with a 2.2-fold increased likelihood of reporting moderate to severe depression. Addressing period poverty in refugee settings is crucial for mitigating depression risks and enhancing overall well-being.

## Introduction

As a global crisis, poverty affects 1.2 billion people worldwide, limiting their access to fundamental necessities such as food, water, and shelter [1]. Yet, in the broader landscape of poverty, there is an often-overlooked aspect that profoundly impacts the lives of menstruators worldwide: period poverty [2]. Period poverty is defined as insufficient access to menstrual hygiene products and basic sanitation facilities alongside inadequate menstrual education [3], it represents a silent struggle experienced by menstruators around the world. Approximately 500 million menstruators express a deficiency in the resources necessary to handle their menstruation [4]. Initially thought to be confined to low and middle-income countries (LMIC), period poverty extends its reach to menstruators with low incomes in high-income countries as well. For instance, in the United States, one in five menstruators experiences period poverty [5]. In addition, 40% of girls in the United Kingdom used toilet paper due to the unaffordability of proper sanitary products, emphasizing the widespread nature of this issue [6].

Period poverty imposes an array of challenges on menstruators at multiple levels. Menstruators with unmet menstrual needs may resort to using unhygienic materials, increasing their risk of urogenital infections [7, 8]. In addition, previous studies revealed high rates of school and work absenteeism due to menstruation [9–11]. Period poverty also has significant emotional and mental impacts on menstruators, leading to feelings of isolation, loneliness, and anxiety, especially among young girls [12]. In a study conducted on college-aged girls in the United States, girls reporting difficulties obtaining menstrual products each month were more likely to report moderate/severe depression [13]. In another study conducted in France, nearly half (49.4%) of menstruators with period poverty reported depressive symptoms, and 40% reported anxiety symptoms [14].

According to United Nations High Commissioner for Refugees (UNHCR), Jordan is the second-largest refugee host globally, with 730,000 registered refugees from multiple nationalities [15]. In addition, there are over two million Palestinian refugees registered with United Nations Relief and Works Agency for Palestine Refugees in the Near East (UNRWA) in Jordan [16]. In such vulnerable settings, the management of menstrual hygiene is particularly challenging, with limited resources, infrastructure, and support. The stigma, embarrassment, and taboos surrounding menstruation further exacerbate the difficulties faced by the displaced population [17]. Despite the urgency of this issue, period poverty remains overshadowed by broader challenges facing displaced menstruators. Our study aims to bridge this gap by focusing on assessing the prevalence of period poverty among refugee menstruators living in camps in Jordan and investigating the association between period poverty and depressive symptoms. The results are expected to inform public health interventions and policies aimed at improving the well-being of this vulnerable population.

## Methods

### Study design and participants

This study employed a cross-sectional methodology to investigate the relationship between period poverty and depressive symptoms among refugee menstruators living in Jordanian camps. The study included menstruators residing in camps in Jordan who were between the age range of post-menarche and pre-menopausal, and who willingly and knowingly gave their agreement to participating in the survey. Menstruators who did not have menstrual periods in the last six months, those who were pregnant or postmenopausal, and those whose data was incomplete were all deemed ineligible.

With 80% statistical power, a 95% confidence interval (CI), and a 5% margin of error, at least 384 individuals were required. This computation is based on the usual method for estimating sample size in observational research [18] and using the Epi Info and accounting for a 95% confidence interval and a 5% margin of error [19].

### Data collection survey tool

Several methods were used to collect the data including face-to-face interviews, an anonymous digital questionnaire using Google forms on social media platforms and shared with menstruators’ official organizations in the different camps. To make sure that the study was comprehensive, the questionnaire for illiterate menstruators was administered by a trained person, and we assured that the participant could understand the questioner. Data on socio-demographic characteristics, menstrual hygiene behaviors, and depressive symptoms were collected. The following socio-demographic variables including age, age at marriage, age at menarche, marital status, children of number, educational level, parental education level, monthly income, employment, and health insurance, were collected.

Period poverty was assessed using two key questions that were adopted from a previous study after translation to Arabic and back translation into English to validate the accuracy of the translations: “In the past 12 months, have you struggled to afford menstrual products (such as sanitary pads or tampons)?” Those who responded “yes” were then asked, “Do you struggle to afford menstrual products every month?” [13]. This allowed for the classification of participants into three groups: participants who stated that they experienced period poverty every month, participants who stated that they experienced period poverty in the past year but not on a monthly basis, and those who stated that they never experienced period poverty [13]. These questions aimed to capture both the affordability and frequency of struggles with menstrual products among participants.

The study used the PHQ-9, a screening instrument consisting of 9 questions specifically created for evaluating depression, to determine the frequency of various symptoms experienced over the past two weeks. These symptoms encompass anhedonia, depressed mood, sleep disturbances, fatigue, changes in appetite, feelings of inadequacy, difficulty concentrating, clumsiness or restlessness, thoughts of self-harm, and suicidal ideation. Respondents scored the distress level caused by each symptom over the last two weeks using a scale with four options: 0 for “not at all,” 1 for “several days,” 2 for “more than half of the days,” and 3 for “nearly every day.” The cumulative score, ranged from 0 to 27, with scores falling into categories including normal, mild, moderate, moderately severe, and severe depression for scores between 0 and 4, 5–9, 10–14, 15–19, and 20 or above, respectively. The validated Arabic version of PHQ-9 was used [20]. The results of the Cronbach’s alpha test in our study showed a value of 0.833 using the whole study sample.

Eight different questions were adopted from the 4-point Likert menstrual practice needs scale (MPNS-36) questionnaire, which focuses on menstrual hygiene practices and environments and the responses included never, sometimes, often, and always [21]. Questions number 2, 4, 7, 29–33 were adopted and the reuse needs score was calculated using question 29–33 as detailed in the MPNS-36 scale manual. This score, based on participants’ responses to questions 29–33 of the MPNS-36, assesses the frequency and extent of women’s need to reuse menstrual hygiene products. Specifically, questions included access to water for soaking or washing menstrual materials (question 29), availability of basins for soaking or washing (question 30), ability to wash menstrual materials when desired (question 31), availability of soap for washing (question 32), and ability to dry materials when desired (question 33). The score ranges from 0 to 3, where 0 represents ‘never’ having enough resources (such as water, basins, soap, or drying facilities) to properly manage menstrual materials, and 3 represents ‘always’ having enough resources. The survey was translated by a bilingual healthcare professional with relevant clinical and research expertise into Arabic and then back translated to ensure quality and consistency. The cronbach’s alpha calculated was 0.875 for the reuse needs scale using the whole study sample.

An expert panel of consultant gynecologists at Jordan University Hospital and the research team examined the questionnaire’s face and content validity to ensure its comprehensiveness, accuracy, and that the questions covered the data needed to assess the study’s goal. A pilot study was conducted on a convenience sample of 22 participants who met the inclusion criteria but were not included in the study to ensure that the items’ language was clear, easy to understand, and culturally acceptable and the Cronbach’s alpha was calculated using the data from the pilot study and was excellent (> 0.9).

### Ethical consideration

Prior to the initiation of data collection, the research followed ethical criteria and received approval from the the Obstetrics & Gynecology at Jordan university hospital and IRB at University of Jordan. To ensure comprehension and voluntary participation, the consent process was conducted in a culturally sensitive manner, with trained researchers explaining the study objectives, procedures, potential risks, and benefits in simple language. Participants were assured of their right to refuse or withdraw from the study at any time without consequences. In addition, no personal information was collected and securely stored all data in compliance with data protection regulations. Informed written consent was obtained from the patients.

### Statistical analysis

SPSS version 26.0 (Chicago, USA) was used for statistical analysis. Variability analysis in the form of the mean ± standard deviation was used to describe age and other continuous variables. Standard descriptive statistical parameters were calculated for sociodemographic characteristics, and responses to questions were reported as counts (frequencies). Percentage values were calculated at the study level. The reliability of the questionnaires was computed via Cronbach’s alpha. For the analyzing the relationship between categorical variables, such as demographic characteristics and period poverty status, Chi-squared test or Fisher’s exact test depending on whether the assumptions of the Chi-squared test are met. For continuous variables, such as age, monthly income, and PHQ-9 scores, an independent sample t-test was used to compare means between two groups, and One Way Analysis of variance (ANOVA) followed by post hoc (LSD) to compare means among multiple groups was used to compare values between the sociodemographic and three groups of period poverty and presented the data as the mean ± standard deviation. Binary logistic regression models were utilized to investigate the relationship between period poverty and mental health outcomes, adjusting for potential confounding variables. Variables with a *p*-value less than 0.1 on univariate analysis were included in the regression models to control for their effects and presented the data as odds ratio (OR) and its associated confidence interval (CI). A *P*-value of < 0.05 was considered statistically significant.

## Results

### Demographic characteristics

The study included a diverse sample of 386 refugee menstruators living in Jordanian camps. Participants’ ages ranged from 13 to 55, with a mean age of 32.43 ± 9.95. Almost two-thirds (65.9%) of participants were married, and the average age at marriage was 20.44 ± 4.21. The mean number of children was 3.67 ± 1.98. Participants had a mean age at menarche of 13.5 ± 1.66. Around one-third of the participants (36.0%) had a higher degree of education. The mean monthly income was 307.41 ± 194.11 Jordanian Dinars (JODs), and this income was shared by a range of 1 to 27 family members. Table 1 shows the demographic characteristics of the study population.

**Table 1:** Characteristics of participants in the study

Variables		Mean ± SD	*N* (%)
Age		32.43 ± 9.95	
Age at marriage		20.44 ± 4.21	
Age at menarche		13.50 ± 1.66	
Marietal status	Married		253 (65.9)
	Single		104 (27.1)
	Divorced		18 (4.7)
	Widowed		9 (2.3)
Children number		3.67 ± 1.98	
Last menstrual period	< 1 month		331 (85.8)
	1–3 months		41 (10.6)
	3–6 months		14 (3.6)
Education	No education		14 (3.6)
	Primary school		104 (26.9)
	Secondary school		129 (33.4)
	Higher education		139 (36.0)
Mother education	No education		115 (29.8)
	Primary school		124 (32.1)
	Secondary school		79 (20.5)
	Higher education		68 (17.6)
Father education	No education		88 (22.9)
	Primary school		132 (34.3)
	Secondary school		100 (26.0)
	Higher education		65 (16.9)
Monthly income		307.41 ± 194.11	
Employment	Unemployed		298 (77.2)
	Part time		41 (10.6)
	Full time		45 (11.7)
	Retired		2 (0.5)
Health insurance	No		201 (52.1)
	Yes		185 (47.9)
Reuse needs score		2.01 ± 0.92	
Reuse material	No		110 (28.5)
	Yes		276 (71.5)
PHQ-9 score		10.92 ± 5.97	
Category PHQ-9	No		61 (15.8)
	Minimal		108 (28.0)
	Moderate		109 (28.2)
	Moderately Severe		74 (19.2)
	Severe		34 (8.8)

### Menstrual health and product affordability

A significant proportion of participants reported facing challenges related to product affordability. Problems in purchasing menstrual products at least once over the last year, but not on a monthly basis, were reported by 15.3% of participants. Furthermore, 42.0% reported struggling with menstrual product affordability on a monthly basis.

Upon univariate analysis, it was found that experiencing period poverty was significantly associated with the younger age of marriage, increased child number, lower education level, lower mother education level, lower father education level, being unemployed, decreased monthly income, without health insurance, decreased reuse need score, and increased PHQ-9 score (*p* < 0.05) (Table 2).

**Table 2:** Sociodemographic according to the period poverty and PHQ-9 categories

	No period poverty	Yearly poverty	Monthly poverty	*P* value	No/minimal depression	Moderate/severe depression	*P* value
**Age**	32.3 ± 9.81	30.12 ± 9.56	33.41 ± 10.14	0.093	31.85 ± 10.12	32.88 ± 9.81	0.317
**Age at marriage**	21.26 ± 4.45	19.60 ± 3.77	19.97 ± 4.01	**0.023**	21.16 ± 4.05	19.91 ± 4.25	**0.014**
**Age at menarche**	13.48 ± 1.42	13.31 ± 1.43	13.59 ± 1.95	0.538	13.32 ± 1.49	13.64 ± 1.78	**0.049**
**Marietal status**				0.404			0.166
Married	101 (61.6)	38 (65.5)	114 (70.4)		104 (61.9)	149 (69)	
Single	52 (31.7)	14 (24.1)	38 (23.5)		52 (31)	52 (24.1)	
Divorced	7 (4.3)	3 (5.2)	8 (4.9)		6 (3.6)	12 (5.6)	
Widowed	4 (2.4)	3 (5.2)	2 (1.2)		6 (3.6)	3 (1.4)	
**Children number**	3.35 ± 1.83	3.11 ± 1.99	4.15 ± 2.01	**0.001**	3.44 ± 1.89	3.83 ± 2.03	0.108
**Last menstrual period**				0.080			**0.011**
< 1 month	146 (88.5)	50 (84.7)	135 (83.3)		153 (90.5)	178 (82)	
1–3 months	14 (8.5)	4 (6.8)	23 (14.2)		9 (5.3)	32 (14.7)	
3–6 months	5 (3)	5 (8.5)	4 (2.5)		7 (4.1)	7 (3.2)	
**Education**				**< 0.001**			**< 0.001**
No education	3 (1.8)	2 (3.4)	9 (5.6)		2 (1.2)	12 (5.5)	
Primary school	22 (13.3)	23 (39)	59 (36.4)		30 (17.8)	74 (34.1)	
Secondary school	50 (30.3)	19 (32.2)	60 (37)		51 (30.2)	78 (35.9)	
Higher education	90 (54.5)	15 (25.4)	34 (21)		36 (21.3)	24 (11.1)	
**Mother education**				**0.001**			0.157
No education	35 (21.2)	54 (33.3)	54 (33.3)		44 (26)	71 (32.7)	
Primary school	47 (28.5)	58 (35.8)	58 (35.8)		50 (29.6)	74 (34.1)	
Secondary school	43 (26.1)	29 (17.9)	29 (17.9)		41 (24.3)	38 (17.5)	
Higher education	40 (24.2)	21 (13)	21 (13)		24 (14.2)	21 (9.7)	
**Father education**				**< 0.001**			**0.027**
No education	25 (15.2)	20 (33.9)	43 (26.7)		30 (17.8)	58 (26.9)	
Primary school	46 (27.9)	22 (37.3)	64 (39.8)		53 (31.4)	79 (36.6)	
Secondary school	52 (31.5)	11 (18.6)	37 (23)		50 (29.6)	50 (23.1)	
Higher education	42 (25.5)	6 (10.2)	17 (10.6)		20 (11.8)	18 (8.3)	
**Employment**				**< 0.001**			**< 0.001**
Unemployed	112 (67.9)	49 (83.1)	137 (84.6)		121 (71.6)	177 (81.6)	
Part time job	17 (10.3)	8 (13.6)	16 (9.9)		14 (8.3)	27 (12.4)	
Full time job	34 (20.6)	2 (3.4)	9 (5.6)		33 (19.5)	12 (5.5)	
Retired	2 (1.2)	0 (0)	0 (0)		1 (0.6)	1 (0.5)	
**Monthly income**	379.12 ± 225.53	287.54 ± 139.14	241.3 ± 146.8	**< 0.001**	350.38 ± 214.48	273.56 ± 169.39	**< 0.001**
**Health insurance**				**< 0.001**			**0.018**
No	67 (40.6)	40 (67.8)	94 (58)		76 (45)	125 (57.6)	
Yes	98 (59.4)	19 (32.2)	68 (42)		93 (55)	92 (42.4)	
**Reuse needs score**	2.27 ± 0.92	2.17 ± 0.73	1.74 ± 0.88	**< 0.001**	2.2 ± 0.92	1.88 ± 0.9	**0.005**
**PHQ-9 score**	8.84 ± 5.87	10.76 ± 6.01	13.09 ± 5.29	**< 0.001**		-	

**Note**: Data are represented as mean ± standard deviation or number (percentage). PHQ-9 denotes the Patient Health Questionnaire-9. Depressive symptom categories were defined as follows: no/minimal depression if the PHQ-9 score was less than 10, and moderate/severe depression if the PHQ-9 score was 10 or greater

The reuse of menstrual products was reported by 71.5% of the menstruators in the study. The PHQ-9 scores of those who reused menstrual hygiene items were substantially higher than those of those who did not reuse them (11.45 ± 5.98 vs. 9.59 ± 5.77, *p*-value = 0.006) (Fig. 1). Regarding menstrual hygiene practices, only 53.6% of participants reported having enough menstrual materials to change them as often as desired most of the time or always. In addition, 42.7% faced difficulties obtaining additional menstrual materials when needed. Furthermore, 43.0% of participants most of the time or always expressed concerns about how to acquire more menstrual materials if they ran out.

**Fig. 2:**
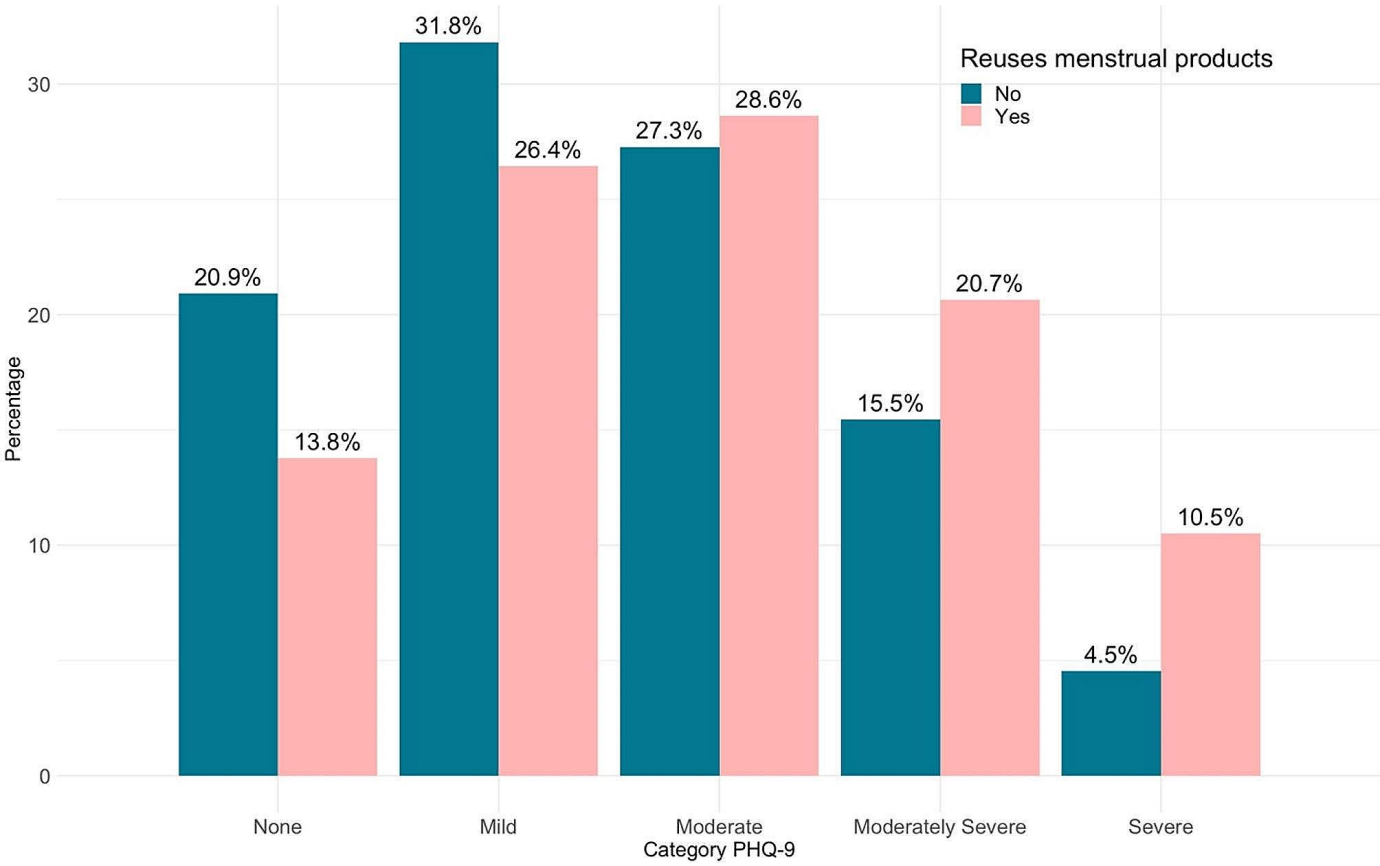
The effect of reusing menstrual products on PHQ-9 score

Access to water and sanitation facilities for menstrual hygiene was also assessed. Among the participants, 48.8% did not always have enough water to soak or wash menstrual materials, and 46.3% did not always have access to a basin for soaking or washing. Also, 32.3% encountered challenges in washing their menstrual materials when desired, and only 48.1% reported sufficient soap always for washing. Table 3 summarizes the answers to the different statements from the MPNS-36 questioner.

**Table 3:** Participants responses toward material and home environment needs and reuse needs items

Statements	Never	Sometimes	Most of the time	Always
Menstrual material sufficiency	56 (14.5)	123 (31.9)	61 (15.8)	146 (37.8)
Access to additional menstrual materials	53 (13.7)	112 [29]	66 (17.1)	155 (40.2)
Concerns about menstrual material availability	99 (25.6)	121 (31.3)	76 (19.7)	90 (23.3)
Adequate water for menstrual material care	32 (12.7)	48 [19]	43 (17.1)	129 (51.2)
Basin access for menstrual material care	28 (10.8)	41 (15.8)	51 (19.7)	139 (53.7)
Timely washing of menstrual materials	28 (10.6)	57 (21.7)	60 (22.8)	118 (44.9)
Soap availability for menstrual material washing	28 (10.5)	57 (21.4)	53 (19.9)	128 (48.1)
Drying accessibility for menstrual materials	35 (13.3)	52 (19.7)	60 (22.7)	117 (44.3)

**Note**: The data is represented as number (percentage)

### Depressive symptoms outcomes

The mean PHQ-9 score was 10.92 ± 5.97, indicating moderate depression on average. The distribution of depression severity levels was as follows: 15.8% none/normal, 28.0% minimal, 28.2% moderate, 19.2% moderately severe, and 8.8% severe.

On post hoc analysis, participants encountering problems purchasing menstrual products over the last year and those experiencing monthly problems had significantly higher PHQ-9 scores than those with no period poverty (10.76 ± 6.01, 13.09 ± 5.29, and 8.84 ± 5.87, respectively, *p*-values = 0.026 and < 0.001, respectively).

Table 4 explores the relationship between period poverty and depression. Results revealed that menstruators experiencing monthly period poverty were 3.948 times more likely to report moderate to severe depression compared to those who had not experienced period poverty (95% CI 2.476–6.295; *P* < 0.001). Upon adjusting for variables that exhibited a *p*-value less than 0.1 in the univariate analysis, consistent findings were observed. Menstruators with monthly period poverty were 2.224 times more likely to report moderate to severe depression than those without period poverty (95% CI 1.069–4.631; *P* = 0.033). The Nagelkerke R-squared was 0.260. The model Chi-square test was *p* < 0.001 and the Hosmer-Lemeshow test was 0.082 indicating a good fit of the model. The overall classification accuracy was 68.9%. Figure 2 illustrates an upward trend in the PHQ-9 scores associated with monthly period poverty.

**Table 4:** Regression model for associations between period poverty and PHQ-9 categories

	No/minimal depression	Moderate/severe depression	Crude odds ratio (95% CI)	*P* value	Adjusted odds ratio (95% CI)	*P* value
No period poverty	97 (57.4)	68 (31.3)	ref		ref	
Yearly poverty	29 (17.2)	30 (13.8)	1.476 (0.812–2.681)	0.202	1.085 (0.387–3.044)	0.877
Monthly poverty	43 (25.4)	119 (54.8)	3.948 (2.476–6.295)	< 0.001	2.224 (1.069–4.631)	0.033

**Note**: The data is represented as number (percentage); adjusted odds ratio (AOR) controlling for all variables that where less than 0.1 on univariate analysis including age at marriage, age at menarche, last menstrual period, education, father’s education, employment, monthly income, health insurance, and reuse needs score

**Fig. 2:**
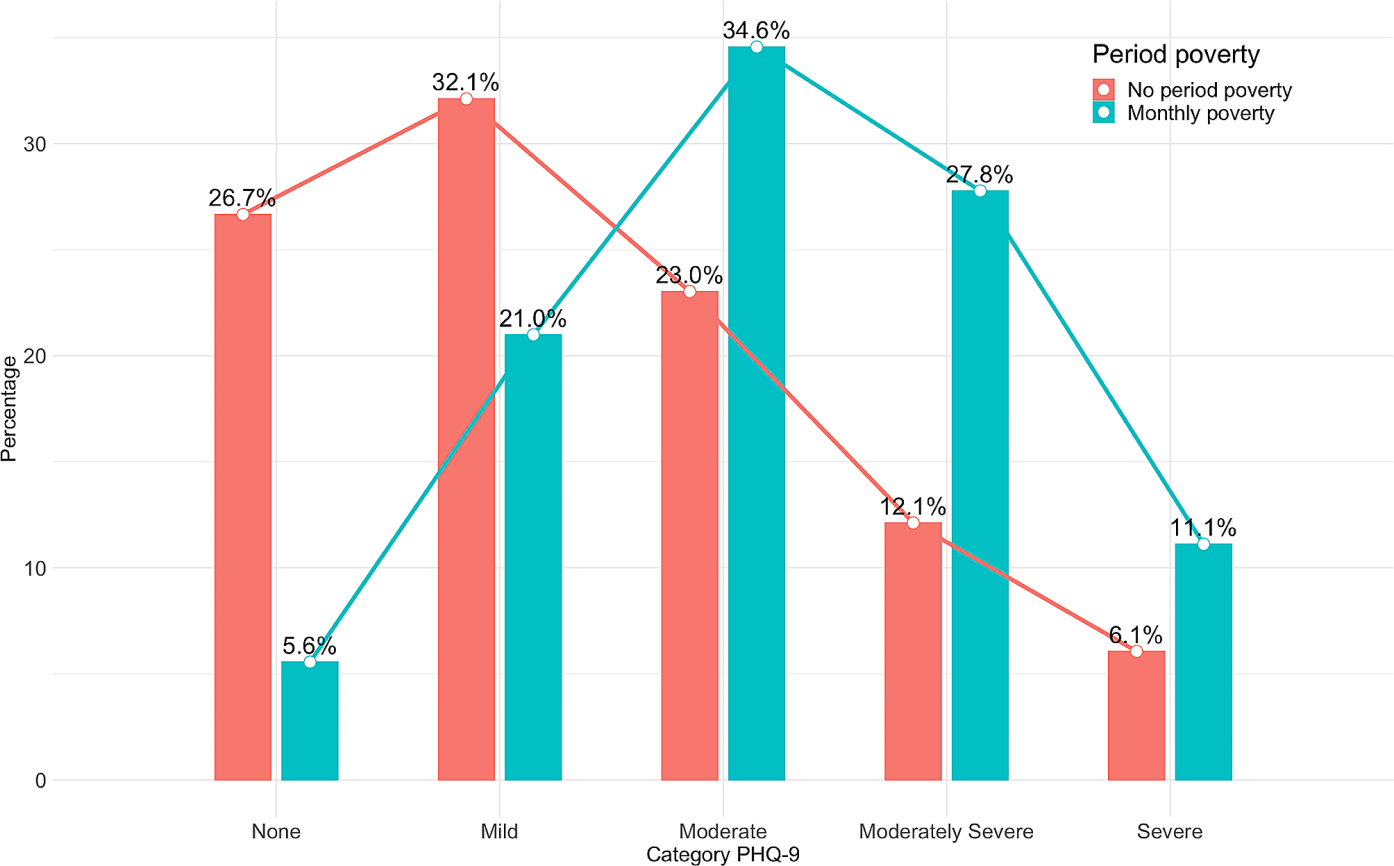
The impact of monthly problems in buying menstrual products on PHQ-9 score

## Discussion

The objective of this study was to find out the prevalence of period poverty, investigate the relationship between period poverty, the practice of reusing menstruation products, and the psychological health of refugee menstruators in Jordanian camps. Our study showed that a substantial proportion of our respondents encountered difficulties related to the accessibility of the menstrual product. This finding was associated with early marriage, higher fertility rates, lower levels of education, unemployment, reduced income, a lack of health insurance, higher levels of depression, and a decreased reuse need score. In addition, more than two-thirds of the participants reported reuse of menstrual products. In our study, the mean total PHQ-9 score indicated an overall moderate depression level among the participants. On multivariant menstruators with monthly period poverty were more likely to report moderate to severe depression than those without period poverty.

Insufficient sanitation may have a significant impact on menstruators’ health, resulting in infections of the reproductive and urinary systems, as well as candidiasis [12]. Our survey found that 42.0% of participants experienced difficulties in affording menstrual hygiene products on a monthly basis. This finding is quite concerning, particularly in comparison to the literature. Cardoso et al. demonstrated that 10% of college-aged females in the United States encounter monthly period poverty [13]. We propose that the variation in the reported prevalence of period poverty might be attributed to disparities in the underlying socioeconomic status of the two groups. This is certainly noteworthy, particularly when considering that the average monthly income of our respondents was 307 JODs and that over 75% of our sample were unemployed. In our study, more than half of the participants did not have health insurance, and according to our results, there was a significant association between health insurance and poverty and depression levels. A prior systematic study indicated that having health insurance increases the likelihood of seeking maternity health services [22]. Another systematic analysis in low and middle income countries, found that greater health insurance coverage appears to promote access to health care facilities and improve health status, emphasizing the necessity of good health care access and health insurance [23].

Based on our results, a significant relationship was found between period poverty and depressive symptoms among refugee menstruators in Jordanian camps. The higher rate of period poverty was shown to be linked to a 2.2-fold greater likelihood of experiencing moderate to severe depression. To the best of our knowledge, no prior studies have assessed the psychological impacts of period poverty among refugee menstruatorswomen; thus, drawing comparisons is challenging. However, our findings align with research that took place in high-income countries. Marí-Klose et al. found that menstruators who reported period poverty are at a higher risk of developing mental health problems [24]. Moreover, based on recent research done in the United States, menstruators who frequently face period poverty are more likely to experience severe depression in comparison to those who have never had period poverty [13]. Jaafar et al. report that period poverty could leads to serious health problems, particularly among teens [2]. These challenges include both physical health and mental health issues such as stress, social isolation, depression, and anxiety [2]. Contributing factors include stigma surrounding menstruation, inadequate toilets, lack of privacy, and insufficient access to clean water, sanitation, and hygienic menstrual products. Furthermore, our results are in line with previous research that has reported a relationship between unaddressed essential needs and an increased probability of experiencing mental health issues. Similar to refugees, individuals who face food and housing insecurities are at increased risk of developing depression, suicidal ideation, and anxiety [25–28].

Many menstruators are compelled to use low-cost, reusable cloth pads that must be washed, dried, and reused. Due to the constrained family income, males exhibit hesitancy in purchasing such high-priced items for their spouses [29]. As a result, menstruators must make adaptations to handle their menstrual hygiene and personal health. Over two-thirds of the participants in our study reported reusing their menstruation products. Prior studies have thoroughly investigated this issue, which has had a significant influence on the mental, physical, and reproductive health of both young menstruators and adolescents [29, 30]. Every individual experiencing menstruation should be entitled to adequate care and fundamental necessities, emphasizing the significance of prioritizing menstrual health as a fundamental human right. Measures such as providing free access to menstruation products for those who menstruate to ensure adequate hygiene represent key actions that should be endorsed by governments worldwide. For instance, the Scottish government was the first to offer unlimited access to hygiene products for all females [31].

Period poverty and poor mental health can have significant long-term consequences on the overall well-being of refugee women. Chronic exposure to inadequate menstrual hygiene can lead to recurring infections and other health issues, potentially exacerbating existing medical conditions and increasing healthcare needs over time. Previous study showed that high prevalence of urinary or reproductive tract infection symptoms among refugee menstruators and its association with poor menstrual hygiene practices [32]. In addition, the lack of hygienic menstrual management and limited access to affordable menstrual products, coupled with insufficient menstrual hygiene education, causes significant discomfort, psychological stress, and financial strain for women and girls [12, 33]. This contributes to period poverty and exacerbates the shame and depression associated with menstruation-related taboos and stigma.

The main strength of our study is that it is the first study to shed light on the relationship between period poverty and depression among an underserved population. However, the authors acknowledge that this study is not without limitations. First, the study is limited by its cross-sectional design, which hinders the establishment of causal links. In addition, the use of self-reported data may introduces the potential for recall bias, as participants may not accurately remember or disclose information about their experiences. Moreover, the study’s generalizability is limited to the refugee group that was sampled in Jordanian camps. Furthermore, it is important to note that our study did not include a specific measure for anxiety. While the PHQ-9 is a robust tool for assessing depression, it does not capture the full spectrum of psychological distress that may include anxiety. Future research should focus on development and evaluation of programs or interventional studies that provide menstrual products and related education. Such interventions are crucial to improving menstrual hygiene management and reducing psychological distress including depression among refugee women, as indicated by our findings.

The findings of this study underscore the critical need for comprehensive interventions and policies to alleviate period poverty and its associated psychological distress among refugee menstruators. To address these challenges, we recommend a multifaceted approach involving improved access to menstrual hygiene products, enhanced education, psychological support, better sanitation infrastructure, and ongoing research. Distributing free or subsidized menstrual hygiene products in refugee camps and urban areas where refugees reside is essential. This effort should involve partnerships with non-governmental organizations (NGOs), humanitarian agencies, and local governments to ensure a steady supply of these products. Additionally, encouraging for policy changes at the national and international levels to include menstrual hygiene products in basic aid packages and recognizing menstrual health as a fundamental human right is also crucial.

Providing accessible mental health services tailored to the needs of refugee menstruators is essential. The cultural context of menstrual hygiene practices among refugee menstruators in Jordan has a substantial impact on their experiences and actions. Menstruation is frequently accompanied by cultural taboos and misconceptions across many societies. These cultural beliefs can result in feelings of shame and embarrassment, which can affect how menstruators handle their menstrual hygiene. Our study highlights the importance of culturally sensitive interventions that respect and incorporate local beliefs and practices. Therefore, implementing culturally sensitive educational programs that focus on menstrual health management is another key recommendation. These programs should provide information on the use of various menstrual products, hygiene practices, and discussing myths about menstruation. Policies should mandate that menstrual health education be included in the curriculum of schools and centers within refugee camps. Finally, providing sanitation facilities in refugee camps, establishing infrastructure development initiatives, and providing water, soap, and disposal systems for menstrual waste.

## Conclusion

the study’s findings shed light on the alarming prevalence of period poverty among refugee menstruators living in Jordanian camps, underscoring the pressing need for targeted interventions to alleviate the challenges they face. The research not only highlights the scarcity of menstrual materials but also emphasizes the profound impact of period poverty on the mental well-being of these vulnerable individuals. The observed association between period poverty and increased depression risk emphasizes the importance of integrating menstrual health support within mental health programs for refugee menstruators. Addressing the multifaceted issue of period poverty requires a concerted effort to ensure that menstruating individuals among refugee populations have unhindered access to essential menstrual products. Moreover, measures such as providing free access to menstruation products for those who menstruate to ensure adequate hygiene represent key actions that should be endorsed by governments worldwide.

## Data Availability

Data sets generated during the current study are available from the corresponding author on reasonable request.
